# Plaque complement activation and cognitive loss in Alzheimer's disease

**DOI:** 10.1186/1742-2094-5-9

**Published:** 2008-03-11

**Authors:** David A Loeffler, Dianne M Camp, David A Bennett

**Affiliations:** 1 Neurology Research Laboratory, William Beaumont Hospital Research Institute, Royal Oak, MI 48073, USA; 2 Rush Alzheimer's Disease Center, Chicago, IL 60612, USA; 3 Department of Neurological Sciences, Rush University Medical Center, Chicago, IL 60612, USA

## Abstract

**Background:**

Complement activation is increased in Alzheimer's disease (AD), but its significance is unclear. The objective of this study was to determine the relationship between complement activation and cognition during the development of AD.

**Methods:**

iC3b, C9, Bielschowsky, and Gallyas staining was performed on aged normal (n = 17), mild cognitively impaired (n = 12), and AD (n = 17–18) inferior temporal gyrus specimens. Plaques were counted in 10× fields with high numbers of Bielschowsky-stained plaques. One-way ANOVA was used to determine between-group differences for plaque counts and measures of cognitive function, and linear regression was used to evaluate global cognition as a function of Bielschowsky-stained plaques. Terms for iC3b- and C9-stained plaques were then added sequentially as additional predictors in a "mediation analysis" model.

**Results:**

Complement was detected on plaques in all groups, and on neurofibrillary tangles only in AD specimens. iC3b, C9, and Bielschowsky-stained plaque counts increased 2.5- to 3-fold in AD vs. other groups (all *p *≤ 0.01). C9 staining was present on some diffuse plaques, as well as on neuritic plaques. Bielschowsky-stained and complement-stained plaque counts were highly correlated, and were negatively correlated with cognitive measures. When the Bielschowsky plaque count was used as a predictor, its correlations with cognitive measures were statistically significant, but when iC3b and C9 plaque counts were added as additional predictors, these correlations were no longer significant. This loss of significance was attributed to multicollinearity, i.e., high correlations between Bielschowsky-stained and complement-stained plaque counts.

**Conclusion:**

Both early-stage (iC3b) and late-stage (C9) complement activation occurs on neocortical plaques in subjects across the cognitive spectrum; contrary to previous reports, C9 is present on some diffuse plaques. Because of high correlations between complement-stained and Bielschowsky-stained plaque counts, quantitative assessment of the extent to which complement activation may mediate the relationship between plaques and cognitive function could not be performed. Additional studies with animal models of AD (if late-stage complement activation can be demonstrated), or possibly a trial in AD patients with an inhibitor of late-stage complement activation, may be necessary to determine the significance of this process in AD.

## Background

Complement activation is a major inflammatory process which assists in the removal of microorganisms and cellular debris and the processing of immune complexes. Complement immunoreactivity is present in the Alzheimer's disease (AD) brain on amyloid-beta (Aβ) – containing plaques, neurofibrillary tangles (NFTs), neuropil threads, and dystrophic neurites [1-4]. Activation proteins generated early in this process function as opsonins, chemokines, and anaphylatoxins [5], some of which have neuroprotective effects [6-8]. Complete complement activation by any of the three complement pathways (classical, alternative, or lectin-mediated) produces C5b-9, the membrane attack complex (MAC), which is neurotoxic [9]. The significance of this process in AD is unclear. Because of the presence of the MAC on plaques and NFTs, it has been suggested that complement activation may contribute to the development of AD [10-12]; the finding that it enhances Aβ toxicity [13] supports this hypothesis. However, conflicting results with regard to this issue have been obtained from animal studies [14,15]. The MAC has not been reported on plaques in the transgenic mouse model of AD used in these studies, the amyloid precursor protein (APP) mouse [16], possibly due to suboptimal binding of mouse C1q to the human Aβ in the plaques of these transgenic mice [17] and/or lack of appropriate antisera for detecting the mouse MAC.

In contrast to late-stage AD, few studies have examined complement activation during the development of the disease. Lue et al. [18] reported a slight increase in plaque-associated MAC staining in the superior frontal gyrus in "high pathology controls," non-demented elderly subjects with extensive AD-type neuropathology. This increase was markedly less than in AD patients although plaque numbers were similar between the two groups. More recently Zanjani et al. [4] found that early complement activation (C4d) on plaques in the temporal cortex increased in parallel with total plaque numbers from very mild to severe clinical AD, whereas the MAC was detected on plaques and NFTs in some very mild cases but consistently only in severe AD. MAC staining on plaques was not observed in non-demented elderly controls. While these reports suggest that early complement activation may increase in the brain during the development of AD, the contribution of this process to the cognitive impairment which characterizes the disease remains unknown. The objective of this investigation was therefore to evaluate the relationship of complement activation to cognition during the development of AD. Inferior temporal gyrus specimens were examined. This region plays a major role in visual perception and recognition [19], and the information it encodes is likely to be involved in other cognitive functions including short- and long-term memory [20]. Atrophy and cholinergic deficits develop here early in the course of AD [21,22].

## Methods

### Brain specimens

Histological sections (6 μm thickness, mounted on Fisherbrand Superfrost/Plus slides, Fisher Scientific, Pittsburgh, PA) of 4% paraformaldehyde-fixed, paraffin-embedded inferior temporal gyrus (Brodman area 20) were obtained from post-mortem brain specimens from non-cognitively impaired, aged normal (AN) subjects (n = 17), individuals with mild cognitive impairment (MCI; n = 12), and subjects with AD (n = 17–18). These groups were based upon clinical diagnosis. Summary statistics (age, educational level, post-mortem interval [PMI]), and global neuropathology score) and measures of cognitive functioning for these subjects are shown in Tables 1 and 2, respectively. The brain specimens were provided by the Rush Alzheimer's Disease Center (hereafter, Rush ADC), Chicago, IL, and were from individuals who had participated in the Religious Orders Study. Details of this study, which was approved by the Human Investigations Committee of Rush University Medical Center, have been published previously [23]. Subjects signed an informed consent and participated in an annual clinical evaluation including medical history, neurological examination, and a battery of cognitive performance tests. These tests assessed a range of clinical abilities commonly affected by aging and AD, including episodic memory, semantic memory, working memory, perceptual speed, and visuospatial ability. Summary measures of each type of cognitive ability were constructed for use in analyses as reported previously [24]. A global measure of cognitive function, based on all individual tests, was also constructed [25]. Based upon their annual evaluation, participants were classified with respect to AD and other common conditions with the potential to impact upon cognitive function. Annual follow-up evaluations were performed in a similar manner by examiners blinded to previously collected data. At the time of death, the available clinical data were reviewed and an opinion was rendered as to the most likely clinical diagnosis. Modified Bielschowsky staining [26] was performed at the Rush ADC and diffuse plaques, neuritic plaques, and NFTs were counted by a board-certified neuropathologist or trained technician as previously described [24]. Neuropathological ratings (Braak, CERAD, and NIA/Reagan scores) were then assigned by the neuropathologist. For statistical analyses, a global pathological score was constructed as described previously [24].

**Table 1:** Subject summary statistics

Group	n	age (yrs)	education (yrs)	PMI (hrs)	Global neuropathology score
AN	17	79.91 ± 1.52	19.41 ± 0.94	7.48 ± 1.08	0.33 ± 0.09
MCI	12	86.71^a ^± 1.93	18.83 ± 1.18	7.67 ± 2.54	0.39 ± 0.12
AD	17–18	89.35^a ^± 1.77	17.78 ± 0.98	7.66 ± 1.27	1.02^b ^± 0.13

AD and MCI subjects were older than AN subjects, but no significant differences were present between groups for educational level or PMI. The global neuropathology scores of the AD group differed from AN and MCI subjects. Data are expressed as means ± SEM. Abbreviations: AD, Alzheimer's disease; AN, aged normal subjects; MCI, subjects with mild cognitive impairment; PMI, post-mortem interval. (^a^*p *< 0.05 vs. AN; ^b^*p *< 0.05 vs. AN and MCI; *data were available from AD specimens for 18 C9-stained sections but only 17 iC3b-stained sections.)

**Table 2:** Subject cognitive measures

Group	Last MMSE	Global cognition	Episodic memory	Semantic memory	Working memory	Perceptual speed	Visuospatial ability
AN	27.76 ± 0.47	0.12 ± 0.05	0.28 ± 0.08	0.21 ± 0.12	-0.07 ± 0.08	-0.10 ± 0.13	0.03 ± 0.12
MCI	27.42 ± 0.81	-0.37 ± 0.16	-0.39 ± 0.21	-0.37 ± 0.21	0.01 ± 0.18	-0.72 ± 0.26	-0.56 ± 0.22
AD	16.89^a ^± 1.99	-1.71^a ^± 0.21	-2.19^a ^± 0.27	-1.44^a ^± 0.24	-1.24^a ^± 0.22	-1.73^a ^± 0.22	-1.16^b ^± 0.20

AD differed from the other groups with regard to last MMSE score, global cognition, episodic memory, semantic memory, working memory, and perceptual speed. Visuospatial ability was different only between AD and AN subjects. Data are expressed as means ± SEM. Abbreviations: AD, Alzheimer's disease; AN, aged normal subjects; MCI, subjects with mild cognitive impairment; MMSE, minimental state examination. (^a^*p *< 0.05 vs. AN and MCI; ^b^*p *< 0.05 vs. AN)

### Staining procedures

Modified Bielschowsky staining [26], Gallyas staining [27], and immunocytochemical staining for iC3b (early-stage complement activation) and C9 (indicative of C5b-9 and representing late-stage complement activation) were performed on serial sections in the Neurology Research Laboratory at William Beaumont Hospital, Royal Oak, MI. Multiple C9 molecules are present in C5b-9, and its presence can be demonstrated by staining for either C9 or C5b-9 [3]; because our preliminary studies revealed staining of higher numbers of plaques in AD specimens with mouse anti-iC3b and goat anti-C9 than with mouse anti-C4d and mouse anti-SC5b-9 (all from Quidel Corp., San Diego, CA), the antibodies to iC3b and C9 were used in subsequent experiments.

### Immunocytochemical staining for iC3b and C9

Procedures were similar to those reported previously [28], with slight modifications. Paraformaldehyde-fixed, paraffin-embedded, Superfrost/Plus-mounted sections were heated for 1 hr at 56ºC. The sections were subsequently deparaffinized and rehydrated through graded ethanol baths, then rinsed in Tris buffered saline (TBS; 0.1 M Tris, 0.85% NaCl, pH 7.6). (This and all subsequent rinses were performed three times at five min intervals.) They were treated for 4 min with 88% formic acid (Mallinkrodt Chemicals, Phillipsburg, NJ), then boiled for 30 min in citrate buffer, pH 6.0 (Antigen Unmasking Solution, Vector Laboratories, Burlingame, CA) and kept in the same buffer for an additional 20 min while they cooled. After rinsing in tap water and then in TBS, the sections were treated with 3% H_2_O_2_/10% methanol in TBS for 30 min to eliminate endogenous peroxidase activity, rinsed in TBS with 0.1% Triton X-100 (hereafter, TBS-T), then treated with TBS with 0.4% Triton, 1% bovine serum albumin (BSA), and 20% normal horse serum (NHS; Vector) for 30 min. The specimens were then incubated overnight at 4ºC with mouse monoclonal anti-human iC3b (Quidel; 1:100 dilution in TBS-0.2% Triton-1% BSA-2% NHS) or goat anti-human C9 (Quidel; 1:5000 dilution in the same buffer). Negative controls, performed for each specimen, consisted of substituting mouse myeloma MOPC-21 (mouse IgG1-kappa of unknown antigenic specificity; Sigma-Aldrich, St. Louis, MO; 1:86 dilution) and normal goat serum (NGS; Vector; 1:5000 dilution) for anti-iC3b and anti-C9 antisera, respectively. After rinsing the sections in TBS-T, biotinylated horse anti-mouse IgG (for iC3b staining) or biotinylated horse anti-goat IgG (for C9 staining) (both from Vector; 1:200 dilution in TBS-T-BSA) was applied at room temperature for 90 min, followed by rinsing in TBS and then incubation in avidin-biotin-horseradish peroxidase conjugate (ABC reagent, Vector; 1:100 dilution in TBS-BSA) for 1 hr. After washing in TBS, the sections were developed with 3,3'-diaminobenzidine (DAB)/H_2_O_2 _with nickel enhancement (DAB Peroxidase Substrate Kit, Vector). iC3b- and MOPC-21-stained sections were developed for 10 min, and C9- and NGS-stained sections were developed for 20 min. Sections were then dehydrated in ethanol baths to xylene and coverslipped with Cytoseal-60 Mounting Medium (Richard-Allan Scientific, Kalamazoo, MI).

### Quantitation of Bielschowsky-stained and complement-stained plaques

In Bielschowsky-stained sections, images from eight 10×-objective fields from areas of high plaque densities were captured with a Nikon Eclipse 80i microscope equipped with a Nikon Digital DXM1200 camera. Nikon ACT-1 (version 2) image analysis software (Nikon USA, Melville, NY) was used to find the same fields in iC3b- and C9-stained serial sections, and additional images were then captured. The numbers of plaques in each of these images (all Bielschowsky images, followed by all iC3b and then all C9 images) were subsequently counted by one investigator (DAL) in a blinded fashion.

### Assessment of neuronal complement staining

Neuronal immunoreactivity in iC3b- and C9-stained sections was scored, in a blinded fashion, on a scale from 0 to 3, with 0 = no complement immunoreactivity, 1 = slight complement immunoreactivity, 2 = moderate complement immunoreactivity, and 3 = extensive complement immunoreactivity. Two observers (DAL and DMC) independently scored the specimens, and then the results were compared. When specimen scores differed (less than 10% of the specimens) the specimens were re-evaluated and a consensus was reached as to the appropriate score.

### NFT complement staining

Blinded observation of the Bielschowsky- and Gallyas-stained sections suggested that few or no NFTs were present in many of the specimens. This was confirmed by NFT counts that were subsequently obtained from the Rush ADC (means ± SEM for NFT counts/mm^2^: AN, 1.35 ± 0.89; MCI, 1.00 ± 0.46; AD, 9.39 ± 2.93). Evaluation of NFT complement staining was therefore limited to specimens with NFT counts ≥ 3/mm^2 ^(three AN, one MCI, and 12 AD specimens). These sections were scanned with the 40× objective by an observer blinded to the diagnosis for each specimen, and a description of NFT complement staining was recorded for each.

### Statistical procedures

One-way analysis of variance (ANOVA) was employed, controlling for subject age, gender, and education, to determine statistical significance between groups for Bielschowsky, iC3b, and C9 plaque counts, global neuropathology, global cognition, and the summary measures for each of the five cognitive areas referred to earlier. Post-hoc testing was performed, when necessary, via Tukey's HSD. Kruskall-Wallace (non-parametric) ANOVA was used to evaluate between-group differences for numbers of iC3b- and C9-stained neurons. Spearman rank correlations were performed to determine the strength of association between each of these variables. For plaque analyses, mean plaque counts from eight 10× fields per specimen were used; a preliminary analysis confirmed the legitimacy of including mean plaque counts, rather than counts from individual fields, in the analysis. Linear regression was employed to model global cognition as a function of global pathology and of Bielschowsky-, iC3b-, and C9-stained plaques, using these terms as individual predictors and global cognition as the outcome. Terms for iC3b-stained plaques, C9-stained plaques, and the combination of iC3b- and C9-stained plaques were then added sequentially as additional predictors in a "mediation analysis" model to determine if they would influence the strength of association between Bielschowsky plaque counts and global cognition. The analysis was subsequently repeated using each of the measures of cognitive function (episodic memory, semantic memory, working memory, perceptual speed, and visuospatial ability) as the outcome. These regression analyses were also adjusted for subject age, gender, and educational level. The significance level for all analyses was set at *p *< 0.05.

## Results

### Age, educational level, PMI, global neuropathology score, and measures of cognitive functioning (Tables 1 and 2)

AD and MCI subjects were older than AN subjects, but no significant differences were present between groups for educational level or PMI. The global neuropathology scores of the AD group differed from AN and MCI subjects, but were similar between the latter two groups. AD subjects differed from the other groups with regard to last MMSE score, global cognition, episodic memory, semantic memory, working memory, and perceptual speed. Visuospatial ability was different only between AD and AN subjects.

### Plaque staining (Figs. 1, 2, 3)

**Figure 1:**
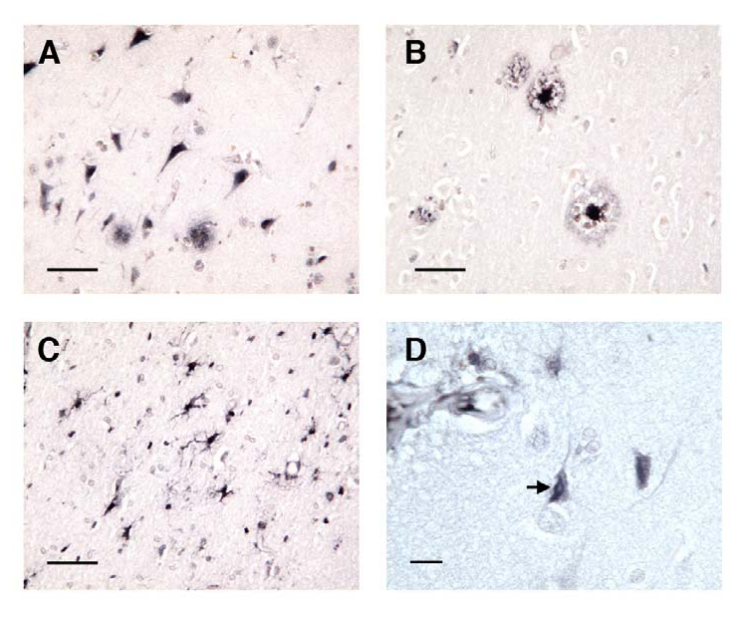
**Complement immunoreactivity in plaques, neurons, and neurofibrillary tangles**. Fig. 1A: iC3b-stained plaques and neurons, MCI specimen; Fig. 1B: C9-stained plaques, AD specimen; Fig. 1C: iC3b-stained glial cells, AN specimen; Fig. 1D, iC3b staining of possible NFT, AD specimen (Figs. 1A – 1C, bar = 50 μm; Fig. 1D, bar = 12.5 μm).

**Figure 2:**
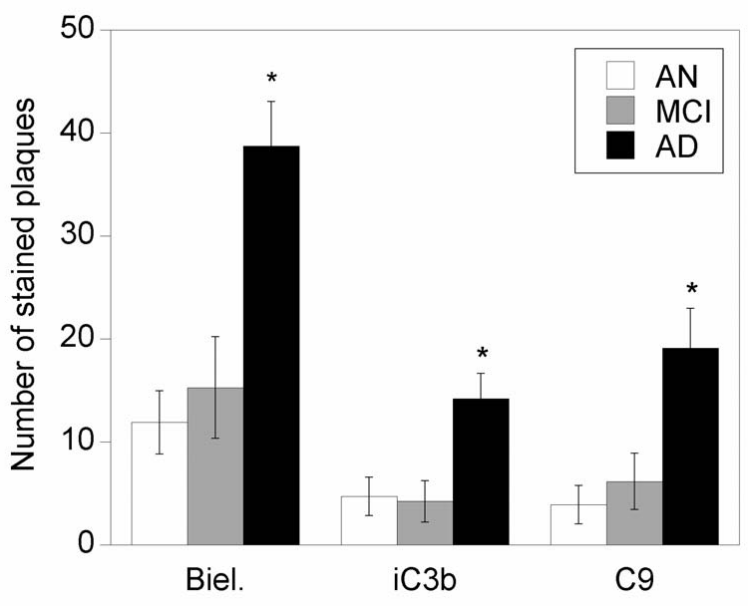
**Bielschowsky, iC3b, and C9 plaque staining**. Means for Bielschowsky, iC3b, and C9 plaque counts in eight 10× fields of high plaque density were significantly increased in AD specimens vs. other groups. Data are expressed as means ± SEM. Abbreviations: AD, Alzheimer's disease; AN, aged normal; Biel, Bielschowsky staining; MCI, mild cognitive impairment. (*p < 0.05 vs. AN and MCI).

**Figure 3:**
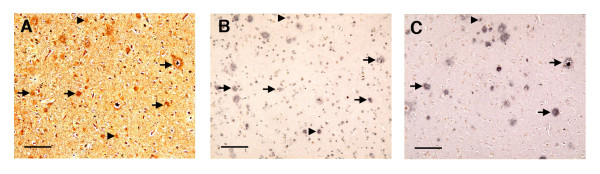
**Alignment of plaques in Bielschowsky, iC3b, and C9-stained sections**. Arrows show neuritic plaques in a Bielschowsky-stained AD specimen (Fig. 3A) that aligned with iC3b (Fig. 3B) and/or C9 plaques (Fig. 3C) in serial sections; arrowheads show diffuse plaques that aligned in the same sections (bars = 100 μm).

Complement staining was detected on plaques, neurons, glia, blood vessels, and, in AD specimens, occasional NFTs (Fig. 1). Bielschowsky, iC3b, and C9 plaque counts were 2.5- to 3-fold higher in AD specimens than in the other groups (*p *= 0.0002, 0.0116, and 0.0130 for Bielschowsky, iC3b, and C9 staining, respectively) (Fig. 2), but were similar between AN and MCI specimens. The numbers of Bielschowsky, iC3b, and C9-stained plaques were highly correlated (pooled specimens from all groups [Spearman rank correlations]: Bielschowsky vs. iC3b, r = 0.88; Bielschowsky vs. C9, r = 0.88; iC3b vs. C9, r = 0.95; all *p *< 0.0001). Assessment of plaque morphology (diffuse vs. neuritic) in Bielschowsky-stained plaques which precisely aligned with complement-stained plaques in serial sections indicated that C9 immunoreactivity was occasionally present on diffuse plaques, as well as on neuritic plaques, in all three groups of specimens (Fig. 3).

### Correlations between plaque staining, cognitive function, and global neuropathology (pooled data)

Bielschowsky, iC3b, and C9 plaque counts were moderately inversely correlated with global cognition and with the five measures of cognitive ability (r = -0.34 to -0.55; all *p *< 0.02). All three types of plaque counts were highly correlated with global neuropathology (r = 0.80 to 0.89; all *p *< 0.0001).

### Influence of Bielschowsky-, iC3b-, and C9-stained plaques on cognition

When Bielschowsky plaques, iC3b plaques, C9 plaques, and global neuropathology were used as individual predictors, statistical significance was present vs. global cognition, episodic memory, semantic memory, working memory, and visuospatial ability as outcomes. These predictors, with the exception of global pathology (*p *= 0.0896), were also significant vs. perceptual speed.

When terms for iC3b-stained plaques, C9-stained plaques, and the combination of iC3b- and C9-stained plaques were sequentially added as additional predictors to the regression analysis model examining the association of Bielschowsky-stained plaques with cognition (using global cognition and each of the five cognitive measures as outcomes), the *p*-values for Bielschowsky-stained plaques and for each of the additional predictors were no longer significant. The one exception to this was episodic memory; when a term for C9-stained plaques was added as an additional predictor, the association between Bielschowsky-stained plaques and episodic memory remained significant (*p *= 0.0258), though far less than when Bielschowsky-stained plaques were used as the only predictor (*p *= 0.0004). The loss of statistical significance for the association of Bielschowsky-stained plaques with cognition, when iC3b- and C9-stained plaque counts were added as additional predictors, was attributed to multicollinearity. Multicollinearity exists, and is irrespective of sample size, when predictor variables are highly correlated with each other [29]; in this case, the high correlations between Bielschowsky and complement plaque counts interfered with the analysis.

### iC3b and C9 NFT and neuronal staining

NFT complement staining was examined in the three AN, one MCI, and 12 AD specimens with NFT counts ≥ 3/mm^2^. Complement immunoreactivity on NFTs was observed only in AD specimens, within apparently intact neurons (Fig. 1D). In contrast, complement staining on neurons (Fig. 1A) was detected in specimens in each of the groups. The extent of this staining was similar between groups, and it was poorly correlated with measures of cognition and with global neuropathology. The numbers of iC3b-stained and C9-stained neurons were highly correlated (r = 0.86 for pooled data).

## Discussion

Complement activation and plaque formation are mutually promoting mechanisms. Aggregated Aβ efficiently binds C1q, activating the classical complement pathway [10], and this process further enhances Aβ aggregation and fibril formation [30]. Plaque-associated complement activation has been suggested to be neurotoxic via bystander lysis [31] and also, indirectly, by chemotactic attraction of microglia [32], which when activated can secrete toxic reactive oxygen species (ROS) [33]. However, because aggregated Aβ alone can also activate microglia [34] and generate ROS [35], the contribution of complement activation to Aβ's neurotoxicity (and to cognitive decline, to the extent that it is associated with plaques) is unclear. We performed regression analysis in an effort to determine the relationship between plaque-associated complement activation and cognition, but the analysis was not informative because of multicollinearity between Bielschowsky-stained and complement-stained plaques. This problem would be present regardless of the numbers of specimens in this study, because statistical power has no bearing on multicollinearity; when two variables are strongly correlated, the strength of this association is independent of the group sizes for the variables [29]. A post-hoc power analysis indicated that this study was highly powered: the range of the power for detecting between-group differences with a 0.05 significance level, based upon the standard deviations within each of the three groups, was 78.4% to 98.3% for iC3b staining, 72.7% to 99.98% for C9 staining, and 98.8% to 99.997% for Bielschowsky staining (data not shown).

The aggregated, fibrillar Aβ in neuritic plaques is considered to be the major factor through which plaques activate complement [10,36], although nonfibrillar Aβ in diffuse plaques may also be able to do so ([37]; however, see Nybo et al. [38]). Previous studies have detected early complement activation proteins on both diffuse and neuritic plaques [1,2,4,12,39], but late-stage complement activation on plaques has been demonstrated primarily, or exclusively, on neuritic plaques [2,3,40,41]. Little or no late-stage complement activation has been reported on plaques in non-demented subjects [3,4]. Our results differ somewhat from these studies in that, in each of the three groups of specimens, plaque staining was detected for C9 (late-stage activation) as well as for iC3b (early-stage activation); in addition, C9 staining was occasionally observed on diffuse plaques, as well as more frequently on neuritic plaques. The slight increases in C9-stained vs. iC3b-stained plaques in MCI and AD specimens are likely to be due to differences in antibody sensitivity (i.e., the polyclonal anti-C9 may detect more plaques than monoclonal anti-iC3b). Detection of C9 on some diffuse plaques suggests that complete complement activation may, in some cases, occur on plaques even in the absence of fibrillar Aβ; whether this contributes to plaque "progression" from diffuse to neuritic is unknown. The similar numbers of Bielschowsky-stained and complement-stained plaques in AN and MCI specimens suggest that neither increased plaque formation nor increased plaque-associated complement activation is required for the development of early cognitive deficits. Our finding of similar plaque counts between AN and MCI subjects is in agreement with a recent study by Petersen et al. [42].

In addition to plaques, complement can be activated in the brain by NFTs [43]. NFT density and distribution are more strongly correlated than plaques with dementia severity [44,45]. There were few or no NFTs in most AN and MCI specimens in this study, in agreement with reports that only in severe dementia are large numbers of neocortical NFTs typically present [46,47]. To determine the relationship between NFT complement activation and cognition, examination of limbic structures such as the hippocampus and entorhinal cortex, in which NFT counts increase early in the disease process [47], would be required. The resulting analysis would still, most likely, be confounded by multicollinearity. Zanjani et al. [4] found little complement staining on NFTs in the entorhinal cortex except in late-stage AD, suggesting that complement activation on NFTs during the development of AD may be minimal.

Neuronal complement staining was present in many of the specimens in this study, but there were no differences between groups for this immunoreactivity and it was poorly correlated with cognitive measures. It is unclear whether this staining indicates complement activation on neurons, neuronal synthesis of native complement proteins, or both. The monoclonal anti-iC3b antibody used in this study is stated by its manufacturer, Quidel, to be iC3b-specific, and to not recognize native C3. This suggests that neuronal iC3b staining is likely to represent complement activation rather than native complement protein synthesis. If neuronal C9 immunoreactivity also represents complement activation (i.e., C5b-9 deposition), then it is unclear why the MAC should be present on normal-appearing neurons. This staining could represent sublytic concentrations of C5b-9, which have been suggested to be neuroprotective [8]. Our findings do not point to complement activation as a direct cause of neuronal injury in AD, although, as stated above, complement-mediated chemotactic attraction of microglia and activation of these cells could indirectly result in neuronal damage due to microglial ROS secretion. Our detection of complement immunoreactivity on normal-appearing neurons differs from an earlier study [48] in which C1q and MAC staining was reported in the AD brain on degenerating neurons. The reasons for these conflicting results are unknown, but may be related to differences in the antibodies employed for complement staining and/or procedures for processing the brain specimens.

Our results demonstrate the limitations of studies with human brain specimens for determining the role of complement activation in AD. Although the extent of this process in the brain correlates strongly with cognitive loss, differentiating its effects from the effects of AD neuropathology on cognition is difficult. Studies in animal models of AD may eventually be of value in resolving this issue if MAC deposition can be demonstrated to the same extent in these models as in AD. A trial in AD patients with an inhibitor of late-stage complement activation such as Eculizumab (Soliris, Alexion Pharmaceuticals) [49], a humanized anti-C5 monoclonal antibody which prevents formation of C5b-9, might also help to resolve this issue.

## Conclusion

Early-stage (iC3b) and late-stage (C9) complement activation occurs on low numbers of plaques in the inferior temporal gyrus in both aged normal individuals and subjects with mild cognitive impairment. Complement staining on plaques is highly correlated with total plaque counts, and both of these parameters are inversely associated with measures of cognitive function. But the strong association between the numbers of complement-stained plaques and total plaque counts results in multicollinearity, which prevents a quantitative assessment of the extent to which complement activation may mediate the association of plaques with cognition during the development of AD.

## Competing interests

The author(s) declare that they have no competing interests.

## Authors' contributions

DAL performed the immunocytochemical staining, plaque counts, assessment of neuronal complement immunoreactivity, and wrote the manuscript. DMC assisted in data collection (capturing of images via the image analysis system) and assessment of neuronal complement staining, generated the figures, performed some of the statistical analyses, and assisted with the writing of the manuscript. DAB assisted in experimental design, obtaining of brain specimens and clinical and post-mortem data, statistical analyses, and provided input during the drafting of the manuscript. All authors read and approved the final manuscript.
